# THE CORRECT IMPLANT CHOICE FOR TRANSTROCHANTERIC FRACTURE IN BRAZIL

**DOI:** 10.1590/1413-785220162406158264

**Published:** 2016

**Authors:** Diogo Fernandes Torquato, André Figueiredo Bordini, Gustavo Ferreira, Edmilson Takehiro Takata, Gustavo Trigueiro, Ricardo Basile

**Affiliations:** 1. Universidade Federal de São Paulo, Escola Paulista de Medicina, Department of Orthopedics and Traumatology, Group of Adult Hip Pathologies, São Paulo, SP, Brazil.

**Keywords:** Hip fractures, Fracture fixation, Femur neck

## Abstract

**Objective:**

To assess the adequacy to the Brazilian population of orthopedic implants used for treatment of proximal femoral fractures.

**Methods:**

The neck-shaft angle of the femur of 101 patients was measured in anteroposterior pelvis radiographs and these measurements were correlated to gender, age, height, weight and ethnicity. In addition, we compared the values of the neck -shaft angle with the angulation of the main implants available in the Brazilian market for the treatment of transtrochanteric fractures.

**Results:**

Of the 101 measurements, an average of 130.9±6.7*°* was obtained, ranging from 112*°* to 150*°*. Correlating these measurements with epidemiological variables, only age was statistically significant.

**Conclusion:**

Most of the analyzed population presented anatomical characteristics that allow the proper use of these implants to treat transtrochanteric fractures, as indicated from the analysis of neck-shaft angles. Nonetheless, 4% of individuals did not fit this pattern and would have required alternative implants. Level of Evidence III, Study of nonconsecutive patients; without consistently applied reference ''gold'' standard.

## INTRODUCTION

The fracture of the proximal femur in the elderly is a public health issue in Brazil. With increasing longevity of the population, the incidence of fractures has also considerably increased.^1^ ^-^ ^3^

For the correct treatment of these fractures, it is necessary to know about the anatomy of the proximal femur, as well as its anatomical variations and of the surgical implants available in this country.^4^

The cervical-diaphyseal angle (CDA) can be measured at the proximal end of the femur, which is larger at birth, approximately 150° ^5^ and gradually decreases with increasing age up to 130±7° on average in the adult population,^2^ ^,^ ^3^ ^,^ ^6^ ^,^ ^7^ and it may reach 120° in the elderly. This angle may also vary according to gender and age.^6^ ^,^ ^8^ ^-^ ^11^

Treatment of transtrochanteric femoral fractures employ implants such as DHS (dynamic hip screw)-type sliding plates and blocked cephalic-spinal rods with predetermined angles. Considering that the implants used in Brazil for the treatment of these fractures are imported from Europe and US, or are domestic products designed based on imported products, it is questionable whether such implants would be appropriate for the profile of the Brazilian population. Therefore, knowing that there are few studies that determined the average CDA of the Brazilian population,^6^ we measured CDA in pelvic radiographs of patients living in São Paulo, SP, Brazil, and correlated the values ​​obtained with epidemiological data. Then, we evaluated the suitability of implants used in the treatment of transtrochanteric fractures in Brazil, based on the data obtained.

## MATERIALS AND METHODS

In the period April-August 2015 we evaluated 101 anteroposterior pelvic radiographs of patients living in the city of São Paulo that were treated at the Adult Hip Pathologies Outpatient of Hospital São Paulo. This is a retrospective study performed by analyzing X-rays from the service's database, without prior submission to the Ethics Committee.

The measurements were performed in hips that had no clinically or radiographically diagnosable pathologies. The study excluded patients with bilateral hip pathologies, previous surgery, proximal femoral fractures and skeletally immature bones.

Radiographs were evaluated in the anteroposterior view of the pelvis where the CDA values of normal hips were measured. The measurement was determined by the angle between the long axis of the femur and the neck axis.

The long axis of the femur was obtained by measuring with a ruler the diameter of the femoral shaft in two different places, finding the midpoint of these lines. The first line was drawn al the level of the smaller trochanter at its most distal point and another line 5cm distal to that one. The axis was determined by joining the two midpoints.^11^

**Figure 1:**
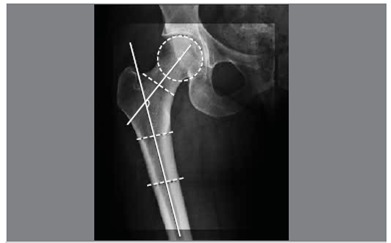
CDA measuremet.

The neck axis was drawn from the center of the femoral head, identified with a goniometer, and a second point located at the mid-thickness of the neck at the basal-cervical level. Drawing a line connecting the two points determines the neck axis. At the intersection between the neck axis and the long axis of the femur there is the cervical-diaphyseal angle (CDA). (Figure 1)

To verify the correlations between measurements of the cervical-diaphyseal angle and data on gender, age, weight, height and ethnicity, the Spearman correlation coefficient was adopted and a significance level of p <0.05 was adopted.

The angles of the implants were compared to the data acquired based on technical materials from different manufacturers available at the manufacturers' websites.

## RESULTS

Of the 101 radiographs analyzed, 42 were from female patients and 59 male patients, 24 were of mixed race, 10 black and 67 white. The ages ranged from 17 to 93 years old, the weight ranged from 48 to 105 kg and the height ranged from 145 to 193 cm. The CDA ranged from a minimum of 112° and a maximum of 150°, 130.9±6.7° on average. Descriptive statistics of these variables can be seen in Table 1.

**Table 1:** Descriptive statistics for the variables angle, age, weight, and height of the patients studied.

Descriptive	Angle (°)	Age (years old)	Weight (kg)	Height (cm)
Mean	130.9	49.9	70.9	164.7
Median	130	50	70	164
Standard Deviation	6.7	19.9	12.9	10.6
Minimum	112	17	48	145
Maximum	150	93	105	193
N	101	101	101	101
IC	1.3	3.9	2.5	2.1

N: number of samples; CI: confidence index

Comparing CDA with the variables ethnicity (p = 0.293), gender (p = 0.782), weight (p = 0.812) and height (p = 0.892), we found no statistically significant differences. (Tables 2 - 4) The variable age, however, showed a statistically significant correlation (p = 0.006). (Table 4)

**Table 2:** Correlation between ethnicity and CDA.

Ethnicity		Mean	Median	Standard Deviation	N	CI	*p* value
Angle (°)	White	130.3	130	6.1	67	1.5	0.293
	Black	130.0	131	5.5	10	3.4	
	Mixed race	133.2	132	8.2	24	3.3	

N: number of samples CI: confidence index p < 0.05

**Table 3:** Correlation between gender and CDA.

	Gender	Mean	Median	Standard Deviation	N	CI	*p* value
Angle (°)	Fem.	131.2	130	7.2	42	2.2	0.782
	Masc.	130.7	130	6.3	59	1.6	

N: number of samples CI: confidence index *p* < 0.05

**Table 4:** Correlation between CDA, age, weight and height.

		Angle (°)
Age	correlation	-27.3%
	*p* value	0.006
Weight	correlation	-2.4%
	*p* value	0.812
Height	correlation	1.4%
	*p* value	0.892

*p* < 0.05

Regarding CDA measured in our sample, we observed that most values ​​were between 126-135° (58.3%), with 20.8% of the measurements above and 20.8% below that range. The distribution of patients according to the CDA values is shown in Table 5.

**Table 5:** Relationship between the proportion of patients and range of CDA in the sample.

Angular variation (°)	112-119	120-125	126-130	131-135	136-140	141-150	Total
Number of patients	4	17	32	27	16	5	101
% Patients	4	16.8	31.7	26.7	15.8	5	100%

## DISCUSSION

According to some authors, the average CDA of human adults is 125±7°.^3^ ^,^ ^4^ ^,^ ^6^ The literature also describes that this angle decreases with age, measuring on average 150° in childhood, 140° in adolescence, 125° in adults, and 120° in the elderly. According to the data collected in this analysis we also observed a statistically significant inverse relationship between age and CDA (p <0.05). (Table 4)

Regarding gender, several papers claim there are angular differences^6^ ^,^ ^11^ ^,^ ^12^ while others report no such difference.^3^ ^,^ ^13^ In the present study we found a slightly greater angle in females, although not statistically significant. (Table 3)

There is a directly proportional relationship between CDA and the length of the femur. Taking as a premise that the greater the individual's height, the largest is the femur's length, it is expected CDA to also be greater.^5^ ^,^ ^6^ We found this direct relationship in our study, although not statistically significant (*p*=0.892). (Table 4)

According to the data collected, there was no statistical significance regarding body weight, corroborating data from the literature.^14^ (Table 4)

When evaluating ethnicity *versus* CDA, we did not observe a statistically significant relationship. We should consider the bias of the patient designating his own ethnicity, which is, therefore, a subjective information. (Table 2)

In order to analyze the adequacy of the implants used in osteosynthesis of transtrochanteric fractures the Brazilian population, we compared their angles to data obtained in this morpho-radiological study.

The main orthopedic implants available for osteosynthesis of transtrochanteric fractures in Brazil are DHS-type sliding plates (Dynamics Hip Screw) with angle ranging from 135° or 150° and blocked cephalic-medullary rods such as PFN (Proximal Femoral Nail^(r)^ Synthes (Solothurn/Switzerland) with 125°, 130° and 135° angles; TFN (Trochanteric Femoral Nail^(r)^ Synthes (Solothurn/Switzerland) with 135° angle; Gamma Nail^(r)^ Stryker (Kalamazoo/USA) with 120°, 125° and 130° angles; PF Targon^(r)^ Aesculap (Tuttlingen/Germany) with 125°, 130° and 135° angles.^15^ ^-^ ^19^ (Table 6) There are also numerous other locally produced rods and plates based on these imported models.

**Table 6:** Angle of the implants assessed.

DHS	PFN	Gamma3	PF Targon	TFN
135°	125°	120°	125°	135°
150°	130°	125°	130°	
	135°	130°	135°	

DHS: Dynamics Hip Screw; PFN: Proximal Femoral Nail; TFN: Trochanteric Femoral Nail;

Of the 101 patients studied, 96% had CDA between 120 and 150°, and were, therefore, compatible with various types of implants available. On the other hand, 4% of our sample did not have compatible implants available, presenting CDA lower than 120°. In such cases, osteosynthesis requires a screw positioning in the upper region of the femoral head, which increases the rate of failure, according to the TAD (tip apex distance) concept recommended by Baumgaertner.^20^

Separately analyzing the implants, we observed that individuals with CDA between 120 and 130° have available a wide variety of devices geometrically suitable to their proximal femur, but this does not occur with 135° or 150° DHS. For 135° angles, the cerebral-spinal rods are suitable as is DHS.

Patients whose femurs have angles greater than or equal to 150° have the option of DHS 150, in addition to DHS 135 positioned in the lower region of the femoral head. The cephalic-spinal rods can also be used, positioning the screw in the lower region of the femoral head, probably without increasing the risk of failure.

## CONCLUSION

The population sample studied had a large ethnic miscegenation with different anthropometric characteristics. Although the majority of this population presents CDA values that allow the proper use of implants for treating trochanteric fracture in Brazil, approximately 4% of the individuals require implants with special angles.
